# Exosomal lncRNA UCA1 modulates cervical cancer stem cell self-renewal and differentiation through microRNA-122-5p/SOX2 axis

**DOI:** 10.1186/s12967-021-02872-9

**Published:** 2021-05-30

**Authors:** Zhihui Gao, Qianqing Wang, Mei Ji, Xiangcui Guo, Li Li, Xiaoke Su

**Affiliations:** 1 Department of Gynecology, Xinxiang Central Hospital, NO. 56 Jinsui Road, Xinxiang, 453000 Henan China; 2 Department of Gynecology, The First Affiliated Hospital of Zhengzhou University, Zhengzhou, 450003 China

**Keywords:** Cervical cancer stem cells, Exosomes, LncRNA UCA1, MicroRNA-122-5p, SOX2, Proliferation

## Abstract

**Background:**

There is growing evidence discussing the role of long non-coding RNAs (lncRNAs) in cervical cancer (CC). We performed this study to explore the impact of exosomal lncRNA urothelial cancer-associated 1 (UCA1) in CC stem cells by sponging microRNA-122-5p (miR-122-5p) and regulating SOX2 expression.

**Methods:**

CC stem cells (CD133^+^CaSki) and exosomes were extracted and identified. The synthesized UCA1- and miR-122-5p-related sequences were transfected into CaSki cells, CaSki cells-derived exosomes were extracted and then co-cultured with CD133^+^CaSki cells. The functional roles of UCA1 and miR-122-5p in self-renewal and differentiation ability of CC stem cells were determined using ectopic expression, knockdown/depletion and reporter assay experiments. An in vivo experiment was performed to verify the in vitro results.

**Results:**

Up-regulated UCA1 and SOX2 and down-regulated miR-122-5p were found in CaSki-Exo. Exosomes promoted invasion, migration, proliferation and restrained apoptosis of CD133^+^CaSki cells. Silencing UCA1 or up-regulating miR-122-5p degraded SOX2 expression, and reduced invasion, migration and proliferation of CD133^+^CaSki cells while advanced apoptosis and suppressed the tumor volume and weight in nude mice.

**Conclusion:**

Our study provides evidence that CaSki-Exo can promote the self-renewal and differentiation ability of CC stem cells while silencing UCA1 or up-regulating miR-122-5p restrains self-renewal and differentiation of CC stem cells.

**Supplementary Information:**

The online version contains supplementary material available at 10.1186/s12967-021-02872-9.

## Introduction

Cervical cancer (CC) is one of the main causes of cancer death in women [1]. High risk human papillomavirus infection [2], and some other exogenous risk factors, such as have sexual relations with several partners, or early sexual behavior, as well as smoking, could contribute to CC risk [3]. The standard primary treatment of CC includes radiotherapy (RT), or radical hysterectomy with pelvic lymph node dissection, or a combination of RT and platinum-based chemotherapy [4]. However, vaginal bleeding is still a familiar result of advanced CC [5]. Consequently, the recognition of novel prognostic markers might be helpful for offering more personalized medical treatment.

LncRNAs are a kind of non-coding RNAs, which are markedly participate in the control of a variety of cell processes [6]. Exosomes are secreted by most cell types, and are considered to play key roles in intercellular communication [7]. It has been explored that CC cells-derived exosomes could enhance angiogenesis in cervical squamous cell carcinoma through delivery of miR-221-3p [8]. Urothelial carcinoma associated 1 (UCA1) is a lncRNA with abnormal expression in a variety of malignant tumors [9]. A study has reported that UCA1 modulates the anti-radioactivity of CC by the hexokinase 2/glycolytic pathway [10]. At present, the role of UCA1 from CC cells-derived exosomes in CC development has been few reported. UCA1 overexpression results in reduced levels of free microRNA-122-5p (miR-122-5p) within the cytoplasm, affecting miR-122-5p to regulate its target mRNAs [11]. miRNAs push forward an immense influence on a series of biological processes which are closely related to cancers, such as differentiation, angiogenesis, proliferation and stress response [12]. As to miR-122-5p, an update report has mentioned its regulatory function in the radiosensitivity of CC cells [13]. Moreover, another study has also presented that lncRNA MIR205HG promotes the growth and development of CC as a competing endogenous RNA (ceRNA) through sponging miR-122-5p [14]. Sox genes encode a class of transcription factors with high mobility and play a key role in organogenesis [15]. It is revealed that sex-determining region Y-box 2 (SOX2) modulates radiation resistance of CC through modulating the hedgehog signaling pathway [16]. Therefore, we speculated that the interaction of UCA1, miR-122-5p and SOX2 in CaSki-Exo might function on the self-renewal and differentiation ability of CC stem cells.

## Materials and methods

### Ethics statement

All animal experiments were in line with the Guide for the Care and Use of Laboratory Animal of the National Institutes of Health. The protocol was permitted by the Committee on the Ethics of Animal Experiments of Xinxiang Central Hospital.

### Cell culture and sorting of CC stem cells (CD133^+^CaSki)

Human CC cell line CaSki was purchased from the Cell bank of the Typical Culture Preservation Committee of the Chinese Academy of Sciences, cultured in dulbecco’s modified eagle medium (DMEM) containing 10% fetal bovine serum (FBS) and placed in a saturated humidity incubator with 37 ℃, 5% CO_2_. The cells were detached with 0.25% trypsin and sub-cultured. CaSki cells in logarithmic growth phase were detached with trypsin, the cell concentration was adjusted to 1 × 10^6^ cells/mL. And then, cells were hatched with CD133 antibody avoiding light for 20 min and the proportion of CD133^+^CaSki cells was detected by flow cytometry. The proportion of CD133^+^CaSki cells in CaSki cells before and after sorting was 2.99% ± 0.47% and 95.42% ± 6.30%, respectively, suggesting the successful sorting.

### Extraction, identification, labeling and grouping of exosomes

Exosomes extraction: CC CaSki cells in logarithmic growth phase were placed in DMEM (Invitrogen, Carlsbad, California, USA) containing 10% FBS without exosomes, and cultured in an incubator for 3 d. The cell supernatant was gathered and centrifuged at 4 ℃ (2000×*g*) for 20 min and the dead cells were removed. And then cells were centrifuged at 4 ℃ (10,000×*g*) for 30 min, and the cell fragments were removed. Next, cells were centrifuged at 4 ℃ (100,000×*g*) for 70 min, and the adherent exosomes were re-suspended and precipitated by PBS, and centrifuged at 4 ℃ (100,000×*g*) again for 70 min. The pellets were re-suspended and precipitated by PBS, centrifuged at ultra-high speed for 2 h, re-suspended and precipitated by PBS, filtered by 0.22 μm filter and then saved at −80℃.

Exosomes identification: (1) CaSki-Exo suspension (20 μL) was placed on the sample copper net for 5 min and dripped with 3% phosphotungstic acid (20 μL) for 5-min staining. Exosomes were dried under a white lamp at 65℃ for 15 min, and the morphology of exosomes was observed under a transmission electron microscope (Hitachi High-technologies Corporation, Tokyo, Japan). (2) The expression of CD63 and CD81 was tested by western blot analysis and the marker protein in exosomes was identified. (3) The size distribution and concentration of exosomes were evaluated by nanoparticle tracking analysis (NTA).

The uptake of CaSki-Exo by CD133^+^CaSki cells was tested by fluorescence labeling: CaSki-Exo suspension (20 μL) was mixed with 1 mL Diluent C solution to prepare for CaSki-Exo working solution which was combined with PKH26 dyeing solution (Sigma-Aldrich Chemical Company, St Louis, MO, USA) for 5 min, added with 2 mL PBS containing 0.1% bovine serum albumin, centrifuged with ultra-high speed (11,000×*g*) for 1 h at 4 ℃ and stored at −80 ℃. Stained CaSki-Exo (10 μL) was added to CD133^+^CaSki culture system for 24 h, fixed with 4% paraformaldehyde, dyed with nuclear 4'-6-diamidino-2-phenylindole (DAPI), observed and photographed under the inverted laser confocal microscope (Olympus, Tokyo, Japan).

Grouping: cells were cultured conventionally in a 5% CO_2_ incubator at 37℃ with 10% FBS-DMEM. The CD133^+^CaSki cells in the logarithmic growth phase were inoculated in the well plate. The control group and CaSki-Exo group (added with 200 μg/mL exosomes) were set, and 5 parallel wells were set up in each group.

### UCA1 and miR-122-5p shuttle experiment

The synthesized sequence siRNA-negative control (NC), siRNA-UCA1, mimic-NC, miR-122-5p mimic, pcDNA-UCA1 + mimic-NC, and pcDNA-UCA1 + miR-122-5p mimic (all purchased from Shanghai GenePharma Co, Ltd, Shanghai, China) were transfected into CaSki cells. After 24 h, the CaSki-Exo were extracted and co-cultured with CD133^+^CaSki cells. A Transwell chamber (0.4 μm) was put in the six-well plates and seeded with 1 × 10^5^ CaSki cells, and 3 × 10^5^ CD133^+^CaSki cells were seeded into the plate for 7 d to establish a co-cultured system.

### Reverse transcription quantitative polymerase chain reaction (RT-qPCR)

The RNA of cells was extracted by RNA extraction kit (Invitrogen). UCA1, miR-122-5p, SOX2, U6 and glyceraldehyde-3-phosphate dehydrogenase (GAPDH) primers were designed by TaKaRa Biotechnology Co. Ltd (Liaoning, China) (Table 1). The RNA was reversely transcribed into cDNA using the PrimeScript RT kit, the reverse transcription system was 10 μL. Fluorescent quantitative PCR was operated in the light of the procedure of the SYBR® Premix Ex Taq™ II. The relative transcriptional levels of target genes were computed by 2^−△△Ct^ method.

**Table 1:** Primer sequence

Gene	Sequence
miR-122-5p	F: 5’ GGGTGGAGTGTGACAATGG 3’
	R: 5’ CAGTGCGTGTCGTGGAGT 3’
U6	F: 5’ CTCGCTTCGGCAGCACATATACT 3’
	R: 5’ACGCTTCACGAATTTGCGTGTC 3’
UCA1	F: 5' CTCTCCTATCTCCCTTCACTGA 3'
	R: 5’ CTTTGGGTTGAGGTTCCTGT 3’
SOX2	F: 5’ GGGAAATGGGAGGGGTGCAAA AGAGG 3’
	R: 5’ TTGCGTGAGTGTGGATGGGGATTGGTG 3’
GAPDH	F: 5’ ACGGCAAGTTCAACGGCACAG3’
	R: 5’ GACGCCAGTAGACTCCACGACA3’

F, forward; R, reverse; miR-122-5p, microRNA-122-5p; UCA1, urothelial carcinoma associated 1; GAPDH, glyceraldehyde phosphate dehydrogenase

### Western blot analysis

The total protein was extracted from the cells and the protein concentration was determined by bicinchoninic acid kit (AmyJet Scientific, Wuhan, Hubei, China). The extracted protein was mixed with the loading buffer and centrifuged after boiling at 95 ℃ for 10 min, separated with 10% polyacrylamide gel electrophoresis, and transferred to membrane. The membrane was sealed with 5% skim milk in tris-buffered saline with tween 20 (TBST) for 1 h, added with primary antibody against SOX2 (1: 1000, Abcam, Cambridge, MA, USA), OCT4 (1: 1000), Nanog (1: 1000) (Cell Signaling Technology, Beverly, MA, USA), CD63 (1: 200), CD81 (1: 200) and GAPDH (1: 1000) (Santa Cruz Biotechnology, Santa Cruz, CA, USA) overnight at 4 ℃. The protein was added with the corresponding secondary antibody (1: 2000, Abcam, Cambridge, MA, USA) for 1 h at 37 ℃ and developed by chemiluminescence reagent. The protein imprinted image was analyzed with ImageJ2x Software (National institutes of health, Maryland, USA).

### Scratch test

CD133^+^CaSki cells (5 × 10^7^ cells/mL) in the logarithmic phase were seeded in the 24-well plates (300 μL/well) with four parallel wells in each group. A sterilized 100 μL pipette tip was adopted for scratching in the 24-well plate and cells were photographed at 0 h and 24 h, respectively to measure the migration distance.

### Invasion and migration experiment

Migration experiment: serum-free DMEM (100 μL) was added to a Transwell upper chamber, and incubated in a 5% CO_2_ incubator for 1 h to activate a polycarbonate membrane. CD133^+^CaSki cells (2 × 10^5^ cells/mL, 100 μL) in the logarithmic growth phase, together with serum-free DMEM were added to the upper chamber, and 600 μL DMEM containing 20% FBS without exosomes was added to the lower chamber, and the cells were incubated for 24 h. The cells were fixed with methanol for 10 min, dyed with 1% crystal violet staining solution for 10 min, pictured under the microscope in eight visual fields and counted.

Invasion test: all steps were the same as the above migration experiment, except that 100 μL serum-free DMEM was changed to 50 mg/L Matrigel (1: 40, 100 μL).

### Flow cytometry

AnnexinV-fluorescein isothiocyanate (FITC)/propidium iodide (PI) double staining was used to analyze cell apoptosis with the Annexin V-FITC Apoptosis Detection Kit I (556547, BD Biosciences, Franklin Lakes, NJ, USA). The cells in the logarithmic growth phase were seeded on the 6-well plate with 2 × 10^5^ cells/well, cultured for 72 h and amassed. The cells were suspended in the 500 μL binding, mixed with 5 μL FITC and 5 μL PI and incubated for 15 min, and the apoptosis was analyzed by flow cytometry.

### Cell counting kit (CCK)-8 assay

When the cell confluence reached about 80%, the cells were made to a single cell suspension, and 3000 cells/100 μL per well were seeded in 96-well plates and hatched in an incubator. CCK-8 reagent (10 μL, Sigma, St. Louis, MO, USA) was added to each well at 24 h, 48 h and 72 h, respectively. Then the cells were continually cultured for 2 h and then the optical density (OD) value at 450 nm of each well was read by a microplate reader (Beijing Potenov Technology Co., Ltd, Beijing, China). The cell viability curve was drawn with the time point as the transverse coordinate and the OD value as the longitudinal coordinate.

### Tumor xenografts in nude mice

Sixty female mice (SJA Laboratory Animal Co., Ltd., Shanghai, China) aged 4 w and weighed 80–90 g were selected. Mice were fed in a clean laminar flow rack of specific pathogen-free grade barrier system, the temperature was (25 ± 1) ℃, and the relative humidity was 40–60%. Matrigel was diluted with serum-free medium to 50%, and mixed with CD133^+^CaSki cell suspension with a proportion of 1: 1. Cells (2 × 10^6^ cells/200 μL) were injected subcutaneously in nude mice. The general status of the nude mice was observed. The tumor volume (V) was monitored every 5 days. Tumor volume was calculated by measuring tumor length (a) and width (b): V = ab^2^/2. The average volume of subcutaneous tumor was calculated, and the tumor growth curve was drawn. Thirty days after injection, the nude mice were euthanized, the tumor was carefully peeled off, pictured and weighed.

### Fluorescence in situ hybridization (FISH) assay

The subcellular localization of UCA1 in cells was identified by FISH. According to the instructions of Ribo™ lncRNA FISH Probe Mix (Red) (RiboBio Co., Ltd, Guangdong, China), the specific methods were as follows: the slide was put into the 24-well culture plate, and the cells were seeded at 6 × 10^4^ cells/well and grown to 80% confluence. The slide was taken out, the cells were fixed with 1 mL 4% paraformaldehyde after cleaning with PBS. After being treated with protease K, glycine and phthalylation reagent, the cells were added with 250 μL pre-hybrid solution and incubated at 42 ℃ for 1 h. The pre-hybrid solution was absorbed, cells were added with 250 μL UCA1 (300 ng/mL) hybrid solution containing probe and hybridized overnight at 42 ℃. The nucleus was stained with phosphate-buffered saline with Tween (PBST)-diluted DAPI (ab104139, 1:100, Abcam, Shanghai, China), added to the 24-well culture plate, and stained for 5 min. The cells were sealed with anti-fluorescence quenching agent, observed and photographed under a fluorescence microscope (Olympus Optical Co., Ltd, Tokyo, Japan).

### Dual luciferase reporter gene assay

The target relationship between UCA1 and miR-122-5p or miR-122-5p and SOX2 and the binding site between miR-122-5p and UCA1 3’untranslated region (3’UTR) or SOX2 3’UTR were analyzed by bioinformatics software https://cm.jefferson.edu/rna22/Precomputed/. The sequence of UCA1 3’UTR or SOX2 3’UTR promoter region containing miR-122-5p binding site was composed to construct UCA1 3’UTR wild-type (WT) plasmid (UCA1-WT) or SOX2 3’UTR WT plasmid (SOX2-WT). The UCA1 3’UTR mutant type (MUT) plasmid (UCA1-MUT) or SOX2 3’UTR MUT plasmid (SOX2-MUT) was constructed by mutation binding site. The CaSki cells with 70% confluence were transfected with UCA1-WT/UCA1-MUT or SOX2-WT/SOX2-MUT with mimic-NC or miR-122-5p mimic by Lipofectamine 2000. The cells were gathered and lysed 48 h after transfection, and luciferase activity was verified by luciferase detection kit (RG005, Shanghai Beyotime Biotech Co., Ltd., Shanghai, China).

### RNA-pull down assay

The biotin labeled miR-122-5p WT plasmid and miR-122-5p MUT plasmid (50 nM each) were transfected into the cells, respectively. After 48 h, the cells were gathered and cleaned with PBS and incubated with specific cell lysate (Ambion, Austin, Texas, USA) for 10 min. And then, 50 mL sample cell lysate was divided into two groups. The residual lysate was incubated with M-280 streptavidin magnetic beads (Sigma, St. Louis, MO, USA) which pre-coated with RNase-free and yeast tRNA (Sigma, St. Louis, MO, USA) for 3 h at 4 ℃. Then the cells were washed twice with cold lysate, three times with low salt buffer, and once with high salt buffer. An antagonistic miR-122-5p probe was set up as a NC. The total RNA was extracted by Trizol and the expression of UCA1 was tested by RT-qPCR.

### Statistical analysis

All data were analyzed by SPSS 21.0 software (IBM Corp. Armonk, NY, USA). The measurement data were represented by mean ± standard deviation. Comparisons between two groups were conducted by t-test, while comparisons among multiple groups were assessed by one-way analysis of variance (ANOVA). *P* value < 0.05 was indicative of statistically significant difference.

## Results

### Successful extraction of CaSki-Exo

**Fig. 1:**
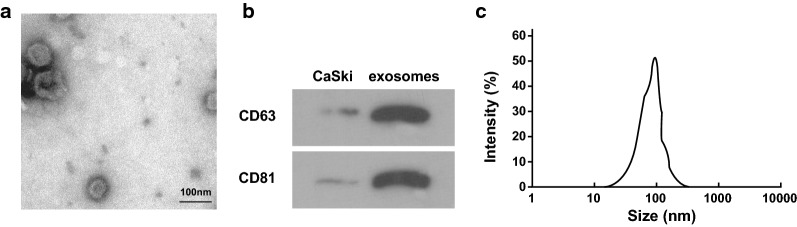
Identification results of exosomes. **a** The morphology of exosomes observed under a transmission electron microscope (× 200,000, 50 nm). **b** western blot analysis detected expression of CD63 and CD81. **c** The size distribution and concentration of exosomes were determined by NTA. Measurement data were depicted as mean ± standard deviation, comparisons between groups were conducted by independent sample *t* test, the experiment was repeated three times

Transmission electron microscope demonstrated that the morphology of CaSki-Exo were in discoid vesicle structure and the diameter was between 30 and 100 nm. CaSki-Exo were coated by complete bilayer lipid membrane. It could seen light central color, thick perimeter staining, and clear edge (Fig. 1a). The results of Western blot analysis revealed that the marker proteins CD63 and CD81 in CaSki-Exo were highly expressed (Fig. 1b) and NTA implied that the diameter of CaSki-Exo was mainly between 40 and 110 nm (Fig. 1c).

### CaSki-Exo advance invasion and migration of CaSki cells

**Fig. 2:**
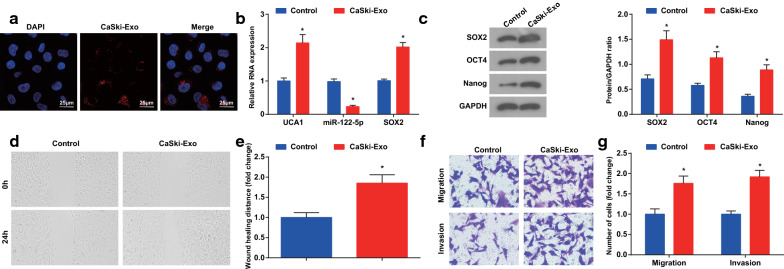
CaSki-Exo promote invasion and migrasion of CaSki cells. **a** The uptake of CaSki-Exo by CD133^**+**^CaSki cells observed by a confocal microscope (× 400, 25 μm). **b** The expression of UCA1, miR-122-5p and SOX2 mRNA detected by RT-qPCR. **c** Expression of SOX2, OCT4 and Nanog protein detected by western blot analysis. **d** and **e** Scratch test tested would healing distance. **f** and **g** Invasion and migration ability of cells tested by Transwell assay. N = 3, * *P* < 0.05 vs. the control group. Measurement data were depicted as mean ± standard deviation, comparisons between groups were conducted by independent sample *t* test

To observe the uptake of CaSki-Exo by CD133^**+**^CaSki cells, CaSki-Exo were co-cultured with CD133^**+**^CaSki cells after labeling with PKH-26 dye, and then a large number of CaSki-Exo with red fluorescence labeling was absorbed by CD133^**+**^CaSki cells under the confocal microscope (Fig. 2a).

RT-qPCR and Western blot analysis reported that the expression of UCA1, SOX2, OCT4 and Nanog was higher while the expression of miR-122-5p was lower in the CaSki-Exo group than the control group (all *P* < 0.05) (Fig. 2b, c).

Scratch test presented that in contrast with the control group, the healing distance of cells in the CaSki-Exo group was longer after 24 h (*P* < 0.05) (Fig. 2d, e). Transwell assay reported that by comparison with the control group, the migration and invasive ability of cells in the CaSki-Exo group were raised (both *P* < 0.05) (Fig. 2f, g).

### Exosomes boost proliferation and restrain apoptosis of CaSki cells

**Fig. 3:**
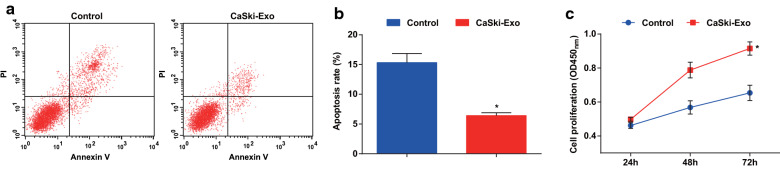
Exosomes promote proliferation and inhibit apoptosis of CD133^+^CaSki cells. **a** AnnexinV-FITC/PI double staining was used to detect the apoptosis of cells in each group. **b** Comparison of apoptosis rate in each group. **c** Comparison of proliferation difference in each group by CCK-8 assay. N = 3, * *P* < 0.05 vs. the control group. Measurement data were depicted as mean ± standard deviation, comparisons between two groups were conducted by independent sample *t* test

AnnexinV-FITC/PI double staining and CCK-8 assay results displayed that the apoptosis rate was lower and the proliferation ability was higher in the CaSki-Exo group than the control group (both *P* < 0.05) (Fig. 3a–c).

### Silencing UCA1 or up-regulating miR-122-5p reduces SOX2 expression and depresses invasion and migration of CD133^+^CaSki cells

**Fig. 4:**
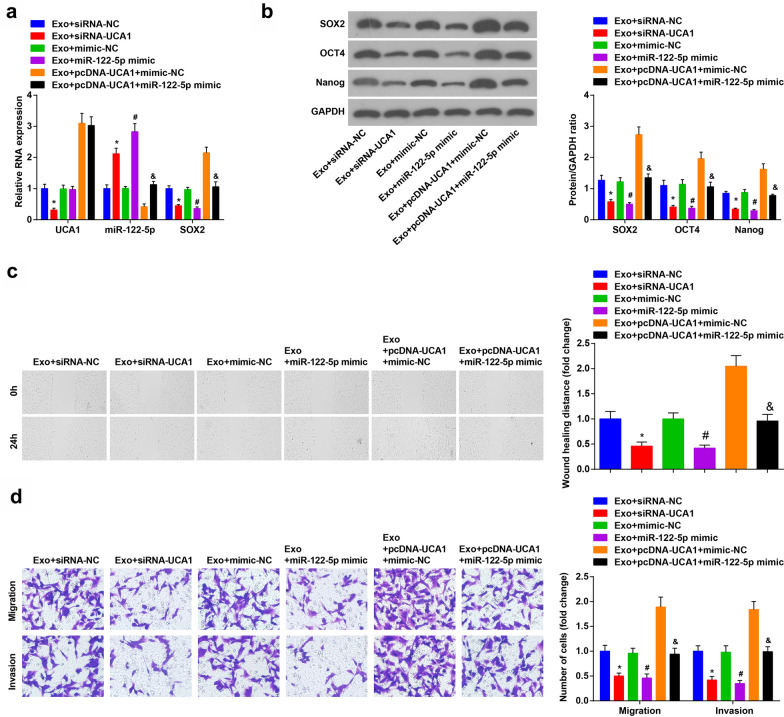
Down-regulated UCA1 or up-regulated miR-122-5p decreases SOX2 expression and the invasion and migration of CD133^+^CaSki cells. **a** RT-qPCR verified UCA1, miR-122-5p and SOX2 mRNA expression. **b** Western blot assay detected SOX2, OCT4 and Nanog protein expression. **c** Effect of UCA1 and miR-122-5p on would healing distance of CD133^**+**^CaSki cells. **d** Effect of UCA1 and miR-122–5 on invasion and migration of CD133^**+**^CaSki cells. N = 3, * *P* < 0.05 vs. the Exo + siRNA-NC group, # *P* < 0.05 vs. the Exo + mimic-NC group. & *P* < 0.05 vs. the Exo + pcDNA-UCA1 + mimic-NC group. Measurement data were depicted as mean ± standard deviation, comparisons among multiple groups were assessed by one-way analysis of variance followed with LSD-*t*

The results of RT-qPCR and Western blot analysis demonstrated that in the Exo + UCA1-siRNA group, the expression of UCA1, SOX2, OCT4 and Nanog was reduced and the expression of miR-122-5p increased versus the Exo + siRNA-NC group (all *P* < 0.05). Compared to the Exo + mimic-NC group, the expression of SOX2, OCT4 and Nanog was degraded, and the expression of miR-122-5p was raised in the Exo + miR-122-5p mimic group (all *P* < 0.05). In contrast with the Exo + pcDNA-UCA1 + mimic-NC group, the expression of SOX2, OCT4 and Nanog were reduced, and the expression of miR-122-5p was enhanced in the Exo + pcDNA-UCA1 + miR-122-5p mimic group (all *P* < 0.05) (Fig. 4a, b).

The CD133^+^CaSki cell invasion and migration were tested. The results presented that the healing distance of cells in the Exo + UCA1-siRNA group was shorter than that of the Exo + siRNA-NC group after 24 h (*P* < 0.05). By comparison with the Exo + mimic-NC group, the healing distance of cells in the Exo + miR-122-5p mimic group was shortened (*P* < 0.05). In relation to the Exo + pcDNA-UCA1 + mimic-NC group, the healing distance of cells in the Exo + pcDNA-UCA1 + miR-122-5p mimic group was decreased (*P* < 0.05) (Fig. 4c).

Transwell assay presented that compared to the Exo + siRNA-NC group, the cell invasion and migration ability of the Exo + UCA1-siRNA group were degraded (both *P* < 0.05). In contrast with the Exo + mimic-NC group, the cell invasion and migration ability of the Exo + miR-122-5p mimic group were reduced (both *P* < 0.05). The invasion and migration of CD133^+^CaSki cells in the Exo + pcDNA-UCA1 + miR-122-5p mimic group were lower than that of the Exo + pcDNA-UCA1 + mimic-NC group (both *P* < 0.05) (Fig. 4d).

### Low expression of UCA1 or high expression of miR-122-5p boosts apoptosis and reduces proliferation of CD133^+^CaSki cells

**Fig. 5:**
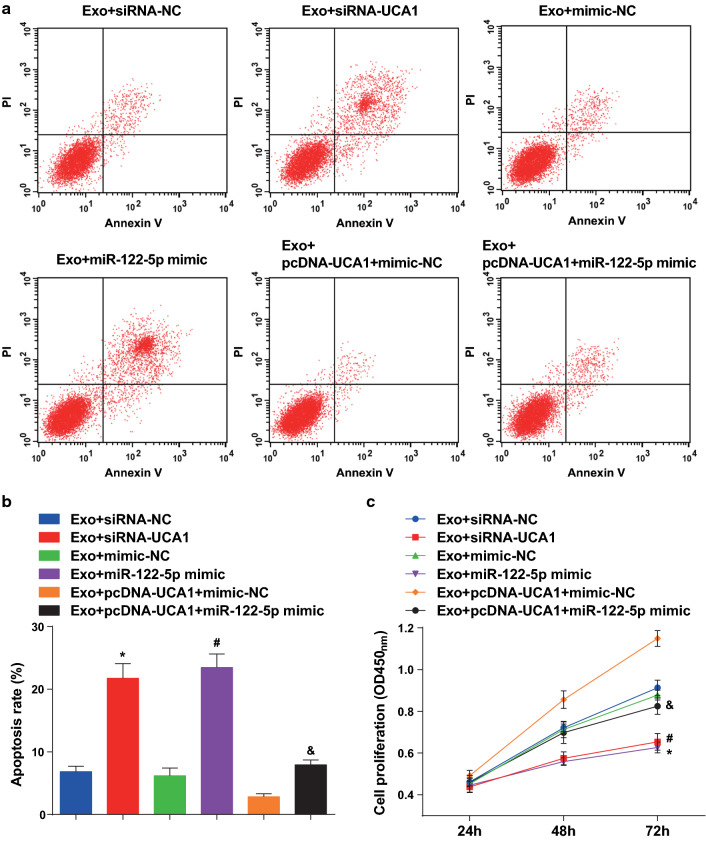
Downregulation of UCA1 or upregulation of miR-122-5p promotes apoptosis and decreases proliferation of CD133^+^CaSki cells. **a** AnnexinV-FITC/PI double staining was used to detect the apoptosis of cells in each group. **b** Comparison of apoptosis rate in each group. **c** Comparison of proliferation difference in each group. N = 3, * *P* < 0.05 vs. the Exo + siRNA-NC group, # *P* < 0.05 vs. the Exo + mimic-NC group. & *P* < 0.05 vs. the Exo + pcDNA-UCA1 + mimic-NC group. Measurement data were depicted as mean ± standard deviation, comparisons among multiple groups were assessed by one-way analysis of variance followed with LSD-*t*

AnnexinV-FITC/PI double staining reported that the apoptosis rate of cells in the Exo + UCA1-siRNA group was higher than that of the Exo + siRNA-NC group (*P* < 0.05). Compared to the Exo + mimic-NC group, the apoptosis rate of cells in the Exo + miR-122-5p mimic group was ascended (*P* < 0.05). In contrast with the Exo + pcDNA-UCA1 mimic-NC group, the apoptosis rate of the Exo + pcDNA-UCA1 + miR-122-5p mimic group was heightened (*P* < 0.05) (Fig. 5a, b). CCK-8 assay demonstrated that by comparison with the Exo + siRNA-NC group, the proliferation of cells in the Exo + UCA1-siRNA group was reduced (*P* < 0.05). In relation to the Exo + mimic-NC group, the proliferation of the cells in the Exo + miR-122-5p mimic group was abated (*P* < 0.05). Compared to the Exo + pcDNA-UCA1 + mimic-NC group, the proliferation of CD133^**+**^CaSki cells was depressed in the Exo + pcDNA-UCA1 + miR-122-5p mimic group (*P* < 0.05) (Fig. 5c).

### Restored miR-122-5p or depleted UCA1 reduces the tumor volume and weight of CC in nude mice

**Fig. 6:**
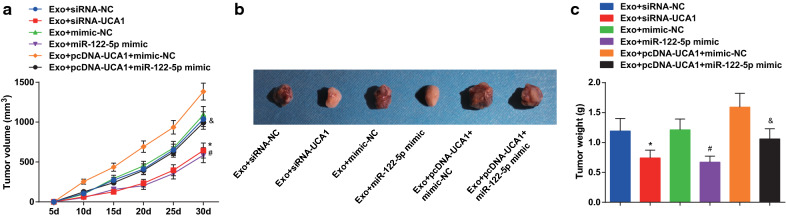
Restored miR-122-5p or depleted UCA1 suppresses the tumor volume and weight of CC in nude mice. **a** The curve of the volume of the tumor after implantation of the cells in each group. **b** Observation of tumor after cell implantation in each group. **c** Tumor weight after cell implantation in each group. * *P* < 0.05 vs. the Exo + siRNA-NC group, # *P* < 0.05 vs. the Exo + mimic-NC group. & *P* < 0.05 vs. the Exo + pcDNA-UCA1 + mimic-NC group. n = 10. Measurement data were depicted as mean ± standard deviation, comparisons among multiple groups were assessed by one-way analysis of variance followed with LSD-*t*

The changes of tumor volume after tumor implantation 5 days and the changes of tumor weight after tumor implantation 30 days in nude mice with CD133^**+**^CaSki cells were detected. The tumor volume and weight were reduced in the Exo + UCA1-siRNA group and the Exo + miR-122-5p mimic group relative to that in the Exo + siRNA-NC group and the Exo + mimic-NC group (all *P* < 0.05). In contrast with the Exo + pcDNA-UCA1 + mimic-NC group, the tumor volume and weight were suppressed in the Exo + pcDNA-UCA1 + miR-122-5p mimic group (both *P* < 0.05) (Fig. 6a–c).

### UCA1 acts as a ceRNA of miR-122-5p to promote SOX2 expression

**Fig. 7:**
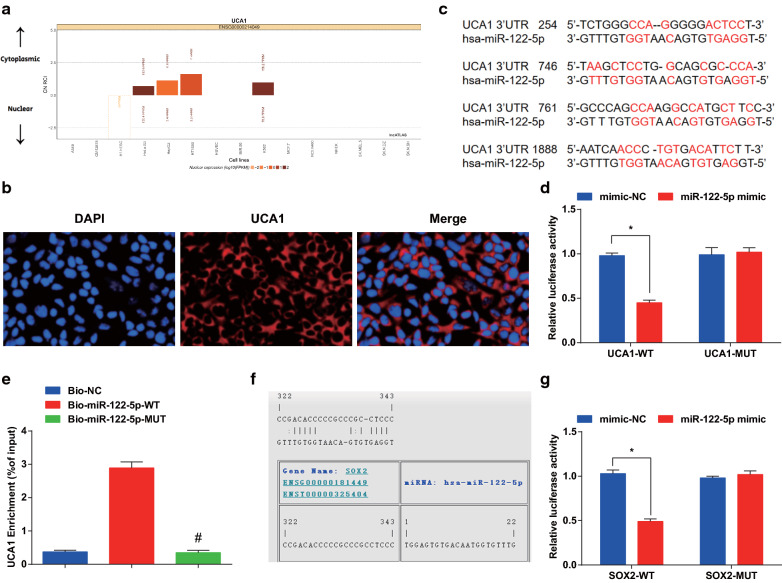
UCA1 acts as a ceRNA of miR-122-5p to affect SOX2 expression. **a** Prediction of subcellular localization of UCA1 by online analysis website. **b** FISH assay verified the subcellular localization of UCA1. **c** Prediction of binding sites between UCA1 and miR-122-5p in RNA22 website. **d** Dual luciferase reporter gene assay detected the binding site of UCA1 to miR-122-5p. **e** RNA pull-down assay verified the enrichment of UCA1 to miR-122-5p. **f** Prediction of the target relationship between miR-122-5p and SOX2 by online website. **g** Dual luciferase reporter gene assay detected the target relationship between miR-122-5p and SOX2. * *P* < 0.05 vs. the mimic-NC group, # *P* < 0.05 vs. the Bio-miR-122-5p-Wt group. Measurement data were depicted as mean ± standard deviation, comparisons between two groups were assessed by independent sample *t* test, and comparisons among multiple groups were assessed by one-way analysis of variance followed with LSD-*t*, the experiment was repeated three times

To investigate the mechanism of UCA1, the http://lncatlas.crg.eu/ website predicted that the UCA1 was mainly distributed in the cytoplasm (Fig. 7a). The secondary structure of UCA1 was observed (Additional file 1: Figure S1). RNA-FISH proved that UCA1 was indeed concentrated in the cytoplasm, suggesting that UCA1 may function in the cytoplasm (Fig. 7b). RNA22 website revealed that UCA1 could combine with miR-122-5p (Fig. 7c). Dual luciferase reporter gene assay demonstrated that the relative luciferase activity of CaSki cells was abated after co-transfected of UCA1-WT and miR-122-5p mimic (*P* < 0.05), while UCA1-MUT and miR-122-5p mimic did not affect the relative luciferase activity of cells (*P* > 0.05), indicating that miR-122-5p may specifically bind to UCA1 (Fig. 7d). RNA pull-down assay reported that the enrichment level of UCA1 in the Bio-miR-122-5p-WT group was higher than that in the Bio-probe NC group (*P* < 0.05), while there was no distinct difference in the Bio-miR-122-5p-MUT group (*P* > 0.05) (Fig. 7e). It is suggested that the UCA1 could be used as a ceRNA to adsorb miR-122-5p, thereby affecting the expression of miR-122-5p.

The results of luciferase activity assay revealed that the relative luciferase activity of CaSki cells was impaired after co-transfection with SOX2-WT and miR-122-5p mimic (*P* < 0.05), while co-transfection with SOX2-MUT and miR-122-5p mimic did not affect the relative luciferase activity of cells (*P* > 0.05). It was indicated that SOX2 was the direct target gene of miR-122-5p (Fig. 7f, g).

## Discussion

CC is the fourth common cancer among women, accounting for almost 7.5 percent of female cancer deaths in the world [17]. It is customarily considered that miR-122-5p unlimited proliferation and malignant progress of CC cells [13]. A previous study has suggested that UCA1 expression inhibited the growth of CC cells [18] and low expression of SOX2 are closely related to poor prognosis in CC patients [19]. As the related mechanisms of UCA1 in CC remains to be excavated, our study was to inquire the effect of exosomal UCA1 from CaSki cells in CC and its inner mechanisms.

In this study, there presented upregulation of UCA1 and SOX2 and downregulation of miR-122-5p in CaSki cells. In line with our results, an analysis demonstrated that UCA1 was elevated in CC [18], and in irradiation-resistant CC cells [10]. Similarly, a previous study has proved that miR-122-5p is lowly expressed in CC cells and conducts as a tumor inhibitor via the rescuing experiments [13]. An article has suggested that the positive rate of SOX2 in CC patients is significantly higher than that in normal controls [20]. Additionally, the finding from our investigation showed that exosomes promoted the migration, proliferation and invasion and inhibited the apoptosis of CaSki cells. HeLa-exo loaded dendritic cells could advance the proliferation of T cells in vitro and induce cytotoxic T-lymphocyte reaction to restrain the growth of CC cells in vitro [21]. The results demonstrated in a recent study highlighting that fetal dermal mesenchymal stem cells-derived exosomes can induce the migration and proliferation secretion of adult dermal fibroblast [22]. The study also showed that UCA1 acted as a ceRNA of miR-122-5p to promote SOX2 expression. A study has revealed UCA1 could act as a ceRNA for miR-122-5p [11], which is in line with our result. Another study has reported that transcription factor SOX4 may be a target gene of miR-122 [23].

Moreover, we demonstrated that silencing UCA1 or up-regulating miR-122-5p would reduce SOX2 expression and depress the invasion, proliferation, migration, boost apoptosis of CD133^+^CaSki cells as well as suppress the tumor weight and volume in nude mice. A study has verified that down-regulation of UCA1 can restrain the invasion and proliferation of CC cells via the expression of miR-206 [18]. Similarly, another paper has demonstrated that silencing of UCA1 decreases the proliferation and migration abilities of colorectal cancer cells in vitro, as well as tumor metastasis in vivo [24], Moreover, in the case of prostate cancer, deficiency of UCA1 decelerates the cancer progression through inhibiting cell proliferation [25]. In addition to that, UCA1 is also an oncogene in gastric cancer that causes enhanced proliferation and migration and reduced apoptosis while depletion of UCA1 restrains tumor progression in vitro and in vivo [26]. As to the role of miR-122-5p in cancer, it is revealed that upregulation of miR-122-5p inhibits the proliferation of gastric cancer SCG7901 cells [27]. Other study also indicated that upregulated miR-122-5p could advance apoptosis, repress cell proliferation, invasion in vitro and inhibit tumor growth in vivo in bile duct carcinoma [28]. Meanwhile, another research has highlighted that raising miR-122 expression is conducive for limiting the malignant biological behaviors of glioblastoma stem cells [29]. Accordingly, overexpressing miR-122-5p in gastric cancer cells has the capacity to impede cellular mobility and invasion, but suppressing miR-122-5p is promoting for tumor metastasis [30].

## Conclusion

Overall, our results suggest that exosomes carrying UCA1 as a ceRNA of miR-122-5p to promote the expression of SOX2, thereby modulating CC stem cell self-renewal and differentiation. Our work identified new clues for further investigating the pathogenesis of CC. Additionally, this study may also offer a theoretical basis for further researches on the mechanisms of UCA1/miR-122-5p/SOX2 in the development of CC.

## Supplementary Information

**Additional file 1: Figure S1.** The secondary structure of UCA1.

## Data Availability

Not applicable.
